# Sickness absenteeism among primary health care workers in Qatar before and during the COVID-19 pandemic

**DOI:** 10.1186/s12995-023-00369-3

**Published:** 2023-03-16

**Authors:** Asma Ali Al-Nuaimi, Sami Abdeen, Muna Abed Alah, Sameera AlHajri, Sandy Semaan, Mohamed Ghaith Al-Kuwari

**Affiliations:** 1 Strategy and Health Intelligence Department, Primary Health Care Corporation, Doha, Qatar; 2 Community Medicine Department, Hamad Medical Corporation, Doha, Qatar; 3 Occupational Health and Safety Department, Primary Health Care Corporation, Doha, Qatar; 4 Collège of Medicine, Qatar University, Doha, Qatar

**Keywords:** COVID-19, Sick leave, Health care worker, Primary healthcare

## Abstract

**Objectives:**

To explore the patterns, trends, nature, and extent of changes in sickness absence among health care workers (HCWs) at the Primary Health Care Corporation (PHCC) in Qatar-during the COVID-19 pandemic compared to previous years and uncover the main associated factors.

**Methods:**

We conducted a retrospective analysis of all sick leaves’ records of PHCC HCWs regardless of their profession from January 2019 till August 2021.

**Results:**

A total of 41,132 sick leaves were taken during the studied period. The majority of HCWs who availed sick leaves were between 30–39 years (45.9%), females (65.1%), and expatriates (65.1%). Compared with pre-COVID-19 (Jan 2019-Feb 2020), Wave 1 of COVID-19 had significantly less incidence of sick leaves per day per 1000 HCWs. While wave 2 had significantly higher incidence of sick leaves compared to both pre-COVID-19 and wave 1. The number of sick leaves per person among female HCWs was significantly higher than that of male HCWs. Moreover, the number of sick leaves per person among locals were about two times the number among expatriate HCWs. Physicians and nurses had significantly lower number of sick leave per person compared to other professions. The rates of sick leaves due to suspected or confirmed COVID-19 infection, back/neck pain and gastroenteritis were significantly higher in the second wave compared to the first wave of COVID-19.

**Conclusion:**

Overall and cause specific sick leave rates among HCWs varied significantly across different periods of the COVID-19 pandemic. COVID-19 related sick leave rate was higher during the second wave compared to first one. By addressing the root causes of sick leaves, it is possible to reduce the burden on HCWs and ensure their continued ability to provide essential care to those in need.

**Supplementary Information:**

The online version contains supplementary material available at 10.1186/s12995-023-00369-3.

## What is already known on this topic

COVID-19 has affected the nature of sickness absence among HCWs and COVID-19 has been linked to increased mental health related sickness absence among HCWs.

## What this study adds

This study is the first one that reports on occupational sickness absence among HCWs in the state of Qatar.

## How this study might affect research, practice and/or policy

This study provides insights into the causes behind sick leaves among HCWs during national emergency. This information can be used to develop strategies to mitigate the impact of sick leaves on HCWs and improve their overall well-being. By addressing the root causes of sick leaves, it is possible to reduce the burden on HCWs and ensure their continued ability to provide essential care to those in need.

## Introduction

The COVID-19 pandemic has overwhelmed health systems worldwide and challenged health care workers (HCWs) with increasing occupational demands while also threatening their health and safety. The increased workload resulted from several factors including, a rise in the number of infected patients and those seeking medical attention during the pandemic, as well as a shortage of staff as many HCWs were infected, isolated due to a history of contact with positive cases, or prevented from work due to personal risk factors. With an increasing workload during infectious outbreaks, HCWs become more prone to stress and complex emotional reactions [1].

The incidence of COVID-19 infection has been higher in HCWs than in most other professions [2, 3]. One systematic review and meta-analysis that included 28 studies reported that 51.7% of HCWs tested positive for COVID-19, with a 15% hospitalization rate and a 1.5% death rate [4]. In Qatar, the rates of COVID-19 infection and hospitalization among HCWs were 10.6%, and 11.6% respectively, according to a study conducted at Hamad Medical Corporation [5,6].

Sick leave can serve as a valuable indicator of the health of the working-age population, providing insights into the overall well-being and productivity. In a large cohort study that compared the total sick leaves in the first trimester of 2020 with that in previous years, it was found that there was an increase in the sick leaves by 116% in March 2020, mostly related to infectious and respiratory diseases [6]. Similarly, in Sweden, the sick-leave rates almost doubled during March and April 2020 compared with the previous year [7]. HCWs sickness absence patterns and rates have also been affected by the pandemic. A study conducted in a single medical center known to have high COVID-19 case load in the United Kingdome (UK) showed significantly higher sickness rates among HCWs during the pandemic compared to the pre-pandemic with significant variation in sickness rates between specialties [8]. The highest levels of sickness absence were seen in medicine, adult, and pediatric emergency departments, while the lowest levels were seen in the intensive care units [8].

Several factors can determine the rates and patterns of sickness absence, such as the health status, age, gender, and psychosocial determinants like personality traits and coping mechanisms. Organizational and work-related factors, such as physical factors like ergonomic factors, psychosocial like job strain, social support, and work schedule also play a role [9–15]. Identifying such factors can help policy and decision makers implement preventive interventions to improve the wellbeing and productivity of workers and reduce sickness absence and costs for employers [16, 17].

The Primary Health Care Corporation (PHCC) serves as the main provider of primary healthcare services in Qatar, catering to both local and expatriate populations. In 2021, the organization recorded over 2 million registered individuals and approximately 3 million physician visits [18]. The services offered by PHCC encompass a broad range of preventive and therapeutic services, including disease screening, immunization, lifestyle counseling, long-term condition management, prenatal care, urgent care, general dental services, pharmacy, and laboratory services [19]. During the COVID-19 pandemic, PHCC assumed a crucial role as a frontline responder, converting some of its health centers into COVID-19 facilities and providing drive-through swabbing services to aid in early detection and contact tracing. The rate of COVID-19 infection among PHCC HCWs was 13.1%, higher compared to rates among HCWs in secondary and tertiary care settings [20]. PHCC implements uniform sick leave policies that apply to all HCWs, regardless of nationality or level of employment. These policies specify the definition of sick leave, the duration of paid sick leave, partial paid and unpaid sick leave, and other related information. HCWs must submit a medical report from an approved medical facility to obtain a sick leave. During the COVID-19 pandemic, PHCC employees were required to increase their working hours to meet heightened demands, yet the sick leave policies remained unchanged, with the exception of adding a special sick leave titled as “COVID-19 sick leave” which is granted to employees with confirmed or suspected COVID-19 infection. The duration of COVID-19 sick leave was determined by national guidelines and varies based on the individual's recovery or quarantine period.

To our knowledge, no studies have been conducted in the Middle East, specifically in Qatar that assessed the impact of COVID-19 pandemic on sickness absenteeism. In this study we aimed to explore the patterns, trends, nature, and extent of changes in sickness absence among HCWs at the Primary Health Care Corporation (PHCC) compared to previous year, and uncover the main associated factors.

## Methods

We conducted a retrospective analysis of all sick leaves’ records of PHCC HCWs regardless of their profession from January 2019 till August 2021. We extracted the sociodemographic characteristics of HCWs (age, gender, nationality), their job characteristic (profession, type of employment, place of work), and sick leave related information such as date, duration, and reason for leave. We compared the sickness absence rates and patterns across five main periods as follows:

- Pre-COVID Pandemic: January 2019 – February 2020
- The first wave of COVID-19 (Wave 1): March 2020 – September 2020
- Post COVID-19 wave 1: October 2020 – January 2021
- The second wave of COVID-19 (Wave 2): February 2021 – April 2021
- Post COVID-19 wave 2: May 2021 – August 2021

### Ethical considerations

Ethical approval was obtained from the research committee of Primary Health Care Corporation with protocol ID (PHCC/DCR/2021/11/064).

### Statistical analysis

The data was analysed using IBM SPSS Statistics for Windows, Version 26.0. Armonk, NY: IBM Corp. Descriptive statistics were presented as frequencies and percentages for categorical variables. To assess differences between proportions of sick leaves between different levels of categorical variables across different time periods we used Chi-square and Fisher exact tests as appropriate. Additionally we used negative binomial regression analysis to analyse the differences and explore the predictors of sick leave rates across different time windows (across years 2019, 2020, 2021; the same month of each year, and the different stages of COVID-19 pandemic), and predictors of the number of days per sick leave and number of sick leaves per person. Incidence risk ratio (IRR) with 95% confidence interval (CI) were calculated to measure the strength of associations. Statistical significance was considered at *p* < 0.05.

## Results

### Sociodemographic characteristics of HCWs who availed sick leaves during the studied period

The study included 6,960 HCWs working with PHCC between January 1, 2019, and August 31, 2021. We found that 41,132 sick leaves were taken during that period. Among the 6,960 HCWs who availed at least one sick leave during the studied period, majority were between 30–39 years (45.9%), females (65.1%) with female to male ratio of 1.9:1. Expatriates accounted for 65.1% of HCWs availing sick leaves, with 1,240 (17.8%) were Indians, and 989 (14.2%) were Filipino. Clinical HCWs accounted for 59.8% of those who availed at least one sick leave, of whom, 2198 (31.6%) were nurses, and 923 (13.3%) were physicians. Upon analyzing the data based on the total sick leaves availed to reflect the frequency of requesting sick leaves per age, gender, nationality, and profession, we found that 42.8% of the 41,132 sick leaves were availed by those 30–39 years old, about three quarters (74.6%) were availed by females, 58%, and 42% were availed by expatriates, and locals respectively, and 52.8% by non-clinical staff (Table 1). The number of sick leaves availed in each month of years 2019–2021 and in different stages of the pandemic by sociodemographic characteristics and reason for sick leave are shown in Supplementary Tables 1–4.

**Table 1:** Number of sick leaves availed in years (2019–2021) by the sociodemographic characteristics and reasons for sick leave

Characteristics		2019 No (%)	2020 No (%)	2021 No (%)	*P*-value^‡§^
**Age categories**	Less than 30	3008 (23.2)	3027 (22.3)	3238 (22.1)	** < 0.001**
	30–39	5495 (42.4)	6025 (44.4)	6082 (41.6)	
	40–49	2991 (23.1)	3153 (23.3)	3597 (24.6)	
	50 or more	1451 (11.2)	1352 (10.0)	1713 (11.7)	
**Gender**	Female	9407 (72.7)	10,247 (75.6)	11,010 (75.3)	** < 0.001**
	Male	3538 (27.3)	3310 (24.4)	3620 (24.7)	
Nationality*	Expatriate	7170 (55.4)	8478 (62.5)	8227 (56.2)	** < 0.001**
	Local	5775 (44.6)	5079 (37.5)	6403 (43.8)	
**Profession**	Allied and Others	8583 (66.3)	8393 (61.9)	9590 (65.6)	** < 0.001**
	Nurse	3092 (23.9)	3803 (28.1)	3600 (24.6)	
	Physician	1270 (9.8)	1361 (10.0)	1440 (9.8)	
**Employment type**	Contracted	87 (0.7)	418 (3.1)	261 (1.8)	** < 0.001**
	Outsourced	344 (2.7)	361 (2.7)	193 (1.3)	
	Permanent	12,379 (95.6)	12,454 (91.9)	13,747 (94)	
	Temporary	135 (1.0)	324 (2.4)	429 (2.9)	
**Reason for sick leave**^¶^	Respiratory related†	4252 (32.9)	3063 (22.4)	1616 (11.3)	** < 0.001**
	Back and/or neck related	1431 (11.1)	1475 (10.9)	2013 (14.1)	
	Gastroenteritis	932 (7.2)	647 (4.8)	793 (5.6)	
	Suspected or confirmed COVID-19	0 (0.0)	2144 (15.8)	2106 (14.8)	
	Contact with confirmed or suspected COVID-19 case	0 (0.0)	108 (0.8)	112 (0.8)	
	Dental related	501 (3.9)	431 (3.2)	464 (3.3)	
	Pregnancy related	308 (2.4)	299 (2.2)	326 (2.3)	
	Mental related	94 (0.7)	87 (0.6)	99 (0.7)	
	Malaise, myalgia or fatigue	221 (1.7)	230 (1.7)	702 (4.8)	
	Tension type headache	302 (2.3)	351 (2.6)	472 (3.2)	
	Others ^¶^	4905 (37.9)	4722 (34.8)	5927 (40.5)	

^*^More than 70 nationalities were reported

†Not labelled as COVID-19 suspected or confirmed

^**‡**^Using Chi square or Fisher exact test as appropriate

^§^This should be interpreted giving the information that the proportions of the number of employees working at PHCC in each year across different levels of the categorical variables (Age categories, Gender, Nationality, Profession and employment type) are almost similar (less than 2% differences between any different combinations), for example the proportions of the number of employees of Allied health and others, nurses and physicians are [56.3, 30.8, 12.9] in 2019, [57.0, 30.4, 12.6] in 2020 and [57.4, 29.9, 12.7] in 2021

^¶^ This include the most common groups of complaints/diagnoses, “Other” category includes any complaint/diagnosis that are not related to those mentioned in the table above (hundreds different complaints/diagnoses) [most commonly: musculoskeletal problems not related to the back and neck, abdominal problems not related to gastroenteritis, surgical related problems, cardiovascular related problems, skin related problems…]. Note that family health issues are not recorded as sick leave and thus not included in our analysis

### Trend analysis of the availed sick leaves

**Fig. 1:**
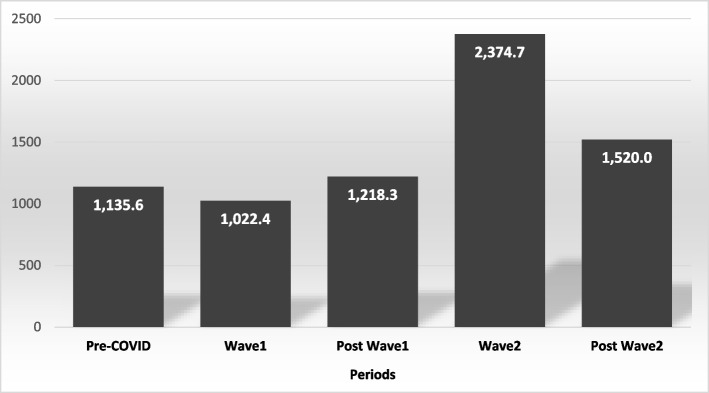
Sick leaves rate (average sick leaves per month) in different stages of the COVID-19 pandemic

In 2019, 12,945 sick leaves were availed by HCWs, October had the maximum number of leaves per month (1,417; 10.9%). A total of 13,557 sick leaves were availed in 2020, with 1563 (11.5%) of them were availed in March 2020. The remaining sick leaves (14,630) were availed up to August 31, 2021, with about one fifth (19.3%) of them being availed in March 2021. As shown in Fig. 1 and Supplementary Table 5, before COVID-19 (from January 2019), a total of 15,898 (38.6%) sick leaves were availed, with an average of 1,135 sick leaves per month, and an average of 1.34 days per sick leave. During the first wave of COVID-19 (March-Sep 2020), 7,157 (17.4%) sick leaves were availed with an average of 1,022 sick leaves per month, and an average of 2.47 days per sick leave (1.83 days/sick leave if we excluded COVID-19 related causes). On the other hand, during the second wave of COVID-19 (Feb-April 2021), 7,124 (17.3%) sick leaves were availed with an average of 2,375 sick leaves per month, and an average of 2.14 days per sick leave (1.54 days/sick leave if we excluded COVID-19 related causes).

Compared with pre-COVID-19 (Jan 2019-Feb 2020), as shown in Table 2, wave 1 had significantly lower incidence of sick leaves per day per 1000 HCWs (IRR 0.77, 95%CI 0.67–0.89, *p* < 0.001). On the other hand, wave 2 had significantly higher incidence of sick leaves compared to both pre-COVID-19 (IRR 1.55, 95%CI 1.27–1.90, *p* < 0.001), and Wave 1 (IRR 2.03, 95%CI 1.63–2.51, *p* < 0.001).

**Table 2:** Differences in the incidence of sick leaves per day per 1000 HCWs between different stages of the COVID-19 pandemic

Stage*	Rate of leaves per day/1000 HCWs	Univariable negative binomial regression	
		**IRR (95%CI)**	***P*-value**
Pre-COVID Pandemic	5.8	1 [Reference]	
Wave 1	4.7	0.77 (0.67–0.89)	** < 0.001**
Post Wave 1	5.4	0.85 (0.72–1.02)	0.078
Wave 2	9.6	1.55 (1.27–1.90)	** < 0.001**
Post Wave 2	6.7	1.126 (0.96–1.34)	0.184

Abbreviations: IRR, Incidence Risk Ratio

^*^ Pre-COVID Pandemic: January 2019 – February 2020, Wave 1: March 2020 – September 2020, Post wave 1: October 2020 – January 2021, Wave 2: February 2021 – April 2021, Post wave 2: May 2021 – August 2021

**Fig. 2:**
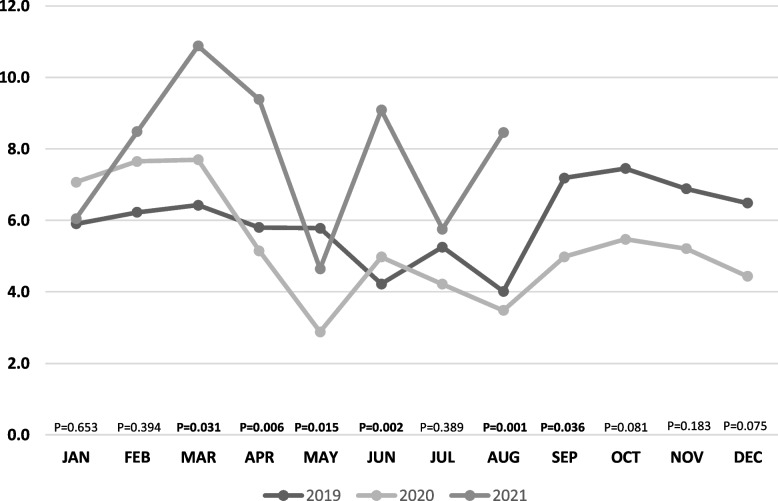
The rate of sick leaves per day per 1000 HCWs in different months in years 2019, 2020, 2021

Comparing the incidence of sick leaves per day per 1000 HCWs between the same months of each year, we found a significant difference between (March–April 2021, June 2021, and August 2021) and the same months in year 2019 (Table 3). Additionally, we found a significantly higher incidence in each of April (IRR 1.82, 95%CI 1.23–2.69, *p* < 0.003), May (IRR 1.61, 95%CI 1.01–2.60, *p* = 0.049), June (IRR 1.83, 95%CI 1.18–2.84, *p* = 0.007), and August (IRR 2.43, 95%CI 1.51–3.92, *p* < 0.001) in 2021 compared to the same months in year 2020. Figure 2 illustrates the rate of leaves per day per 1000 HCWs in different months across years 2019–2021.

**Table 3:** Differences in the incidence of sick leaves per day per 1000 HMCs between the same months of each year (2019, 2020, 2021)

Month	Year	Rate of sick leaves per day/1000 HCWs	Univariable negative binomial regression	
			**IRR (95%CI)**	***P*-value**
**January**	2019	6.0	1 [Reference]	
	2020	7.0	1.20 (0.79–1.82)	0.396
	2021	6.1	1.02 (0.67–1.56)	0.910
**February**	2019	6.3	1 [Reference]	
	2020	7.6	1.23 (089–1.92)	0.367
	2021	8.5	1.36 (0.87–2.14)	0.180
**March**	2019	6.4	1 [Reference]	
	2020	7.7	1.20 (0.80–1.79)	0.382
	2021	10.9	1.69 (1.13–2.53)	**0.010**
**April**	2019	5.7	1 [Reference]	
	2020	5.2	0.89 (0.60–1.31)	0.550
	2021	9.4	1.62 (1.09–2.39)	**0.016**
**May**	2019	5.7	1 [Reference]	
	2020	2.8	0.50 (0.31–0.81)	**0.005**
	2021	4.7	0.80 (0.50–1.31)	0.377
**June**	2019	4.2	1 [Reference]	
	2020	5.0	1.18 (0.75–1.85)	0.471
	2021	9.0	2.15 (1.38–3.37)	**0.001**
**July**	2019	5.3	1 [Reference]	
	2020	4.3	0.80 (0.50–1.28)	0.352
	2021	5.7	1.10 (0.89–0.74)	0.701
**August**	2019	4.0	1 [Reference]	
	2020	3.4	0.87 (0.53–1.42)	0.571
	2021	8.4	2.11 (1.28–3.46)	**0.003**

### Predictors of healthcare worker’s sick leave rate

Using multivariable negative binomial regression analysis, and after adjusting to other variables we found that gender, nationality and profession were significantly associated with the number of sick leaves availed per HCW during the studied period. The number of sick leaves per person among female HCWs was significantly higher than that of male HCWs (IRR 1.47, 95%CI 1.40–1.54, *p* < 0.001). Moreover, the number of sick leaves per person among locals were about two times the number among expatriate HCWs (IRR 2.02, 95%CI 1.91–2.13, *p* < 0.001). On the other hand, physicians (IRR 0.81, 95%CI 0.75–0.87, *p* < 0.001), and nurses (IRR 0.88, 95%CI 0.84–0.93, *p* < 0.001) had significantly lower number sick leave per person compared to other professions (Table 4).

**Table 4:** Predictors of number of leaves availed by HCWs during 2019–2021 using multivariable negative binomial regression

Variable		Average number of sick leaves per person	Negative binomial regression	
			**IRR (95%CI)**	***P*-value**
**Age**		–––––––––-	1.02 (1.01–1.02)	** < 0.001**
**Gender**	Female	7	1.47 (1.40–1.54)	** < 0.001**
	Male	4	1 [Reference]	
**Nationality**	Locals	10	2.02 (1.91–2.13)	** < 0.001**
	Expatriates	5	1 [Reference]	
**Profession**	Allied health and others	7	1 [Reference]	
	Nurse	5	0.88 (0.4–0.93)	** < 0.001**
	Physician	4	0.81 (0.75–0.7)	** < 0.001**

Abbreviations: *IRR * Incidence rate ratio

### Causes of the availed sick leaves

As shown in Table 5, upon analyzing the causes behind sick leaves in different time periods, we found overall that the most common reasons for requesting sick leaves before COVID-19 were respiratory related symptoms (33.6%), back and/or neck pain (10.9%), and gastroenteritis (7%). During the first wave, suspected or confirmed COVID-19 infection, respiratory symptoms/diagnoses (not labelled as suspected or confirmed COVID-19 infection), and back/neck pain were among the top three causes accounting for 20.6%, 18.4%, and 10.6% of the sick leaves availed during the first wave. Similarly, during the second wave of COVID-19, the top three causes were suspected or confirmed COVID-19 (19.4%), back and/or neck pain (12.5%), and respiratory symptoms (not labelled as suspected or confirmed COVID-19) (10.6%). On the other hand, after the second wave of COVID-19, back and/or neck pain came at the top of the list (16.7%), followed by respiratory symptoms (not labelled as suspected or confirmed COVID-19) (10.6%),

**Table 5:** Number of sick leaves availed in different stages of COVID-19 pandemic by reasons for sick leave

Reasons for sick leaves*	Pre-COVID 19 Pandemic		Wave 1		Post Wave 1		Wave 2		Post Wave 2		*P*-value^†^
	**No (%)**	**Number of sick leaves/1000 HCWs**	**No (%)**	**Number of sick leaves/1000 HCWs**	**No (%)**	**Number of sick leaves/1000 HCWs**	**No (%)**	**Number of sick leaves/1000 HCWs**	**No (%)**	**Number of sick leaves/1000 HCWs**	
**Respiratory related**	5352 (33.6)	798.7	1318 (18.4)	185.5	567 (17.9)	77.1	739 (10.6)	88.9	628 (10.6)	87.1	** < 0.001**
**Back and/or neck related**	1734 (10.9)	258.8	757 (10.6)	106.6	570 (11.8)	77.5	873 (12.5)	105.0	985 (16.7)	136.6	
**Gastroenteritis**	1116 (7.0)	166.5	305 (4.3)	42.9	220 (4.5)	29.9	296 (4.2)	35.6	435 (7.4)	60.3	
**Suspected or confirmed COVID-19**	0 (0.0)	0.0	1476 (20.6)	207.8	905 (18.7)	123.1	1351 (19.4)	162.5	518 (8.8)	71.9	
**Dental related**	622 (3.9)	92.8	203 (2.8)	28.6	155 (3.2)	21.1	191 (2.7)	23.0	225 (3.8)	31.2	
**Pregnancy related**	380 (2.4)	56.7	144 (2)	20.3	123 (2.5)	16.7	133 (1.9)	16.0	153 (2.6)	21.2	
**Mental related**	111 (0.7)	16.6	51 (0.7)	7.2	32 (0.7)	4.4	38 (0.5)	4.6	48 (0.8)	6.7	
**Malaise, myalgia, or fatigue**	250 (1.6)	37.3	130 (1.8)	18.3	123 (2.5)	16.7	427 (6)	51.4	223 (3.7)	30.9	
**Tension type headache**	377 (2.4)	56.3	179 (2.5)	25.2	141 (2.9)	19.2	242 (3.4)	29.1	186 (3.1)	25.8	
**Others**	5956 (37.5)	888.8	2594 (36.3)	365.1	2037 (35.3)	277.0	2843 (38.8)	342.0	2679 (43.5)	371.6	

^*^ These include the most common groups of complaints/diagnoses. “Other” category includes any complaint/diagnosis that are not related to those mentioned in the table above (hundreds different complaints/diagnoses) [most commonly: musculoskeletal problems not related to the back and neck, abdominal problems not related to gastroenteritis, surgical related problems, cardiovascular related problems, skin related problems…]. Note that family health issues are not recorded as sick leave and thus not included in our analyses

†Using Chi square test or Fisher exact as appropriate. Which is interpreted as the significance of the differences of the proportions (distribution) of different reasons for sick leaves in different time stages

Further analysis on the most common reasons for sick leaves during different COVID-19 stages showed that the rates of respiratory-related sick leaves per day per 1000 HCWs (that are not labelled as suspected or confirmed COVID-19 infection) were higher during the Pre-COVID-19 stage compared to the other stages (as shown in Table 6). There was no significant difference in the rates between wave 1 and wave 2. Additionally, we observed that the rates of sick leaves due to back/neck pain were significantly higher during wave 2 and post wave 2 stages compared to the Pre-COVID-19 stage. Furthermore, the rates during wave 2 were higher compared to those in wave 1 (IRR 2.35, 95%CI 1.51–3.68, *p* < 0.001). In terms of gastroenteritis-related causes, we found that the rates of sick leaves were significantly lower during wave 1 and the post wave 1 stages compared to the Pre-COVID-19 stage. However, the rate was significantly higher during wave 2 compared to wave 1 (IRR 1.97, 95%CI 1.18–3.29, *p* = 0.009). Finally, we discovered that wave 2 had significantly higher rates of sick leaves due to suspected or confirmed COVID-19 infection compared to wave 1 (IRR 1.88, 95%CI 1.34–2.66, *p* < 0.001).

**Table 6:** Differences in the rates of cause specific sick leaves per day per 1000 HCWs between different stages of the COVID-19 pandemic

Stage	Rate of leaves per day/1000 HCWs	Univariable negative binomial regression	
		**IRR (95%CI)**	***P*-value**
**Respiratory related**			
** Pre-COVID Pandemic**	1.96	1 [Reference]	
** Wave 1**	0.87	0.47 (0.40–0.55)	** < 0.001**
** Post Wave 1**	0.97	0.54 (0.45–0.65)	** < 0.001**
** Wave 2**	1.00	0.63 (0.51–0.78)	** < 0.001**
** Post Wave 2**	0.71	0.39 (0.32–0.47)	** < 0.001**
**Back and/or neck related**			
** Pre-COVID Pandemic**	0.64	1 [Reference]	
** Wave 1**	0.50	0.83 (0.71–0.98)	0.029
** Post Wave 1**	0.64	1.10 (0.90–1.33)	0.352
** Wave 2**	1.18	2.30 (1.87–2.84)	** < 0.001**
** Post Wave 2**	1.11	1.88 (1.56–2.26)	** < 0.001**
**Gastroenteritis**			
** Pre-COVID Pandemic**	0.41	1 [Reference]	
** Wave 1**	0.20	0.47 (0.33–0.66)	** < 0.001**
** Post Wave 1**	0.24	0.55 (0.36–0.83)	**0.005**
** Wave 2**	0.40	0.92 (0.57–1.48)	0.721
** Post Wave 2**	0.49	1.14 (0.75–1.73)	0.537
COVID-19 related*			
** Pre-COVID Pandemic**	0.00	––––	**–––**
** Wave 1**	1.04	1.70 (1.28–2.25)	** < 0.001**
** Post Wave 1**	1.03	1.67 (1.21–2.29)	** < 0.001**
** Wave 2**	1.95	3.20 (2.26–4.52)	** < 0.001**
** Post Wave 2**	0.60	1 [Reference]	

Abbreviations: *IRR * Incidence Risk Ratio

^*^Note that pre-COVID pandemic period was not included in the analysis

## Discussion

Understanding the circumstances and factors affecting sickness absenteeism among HCWs during national emergencies like the COVID-19 pandemic is crucial to maintain a well-functioning healthcare system and to ensure the continuity and quality of healthcare services and the stability of healthcare workforce. In this study, we investigated the patterns, trends, nature, and the extent of changes in sickness absence among HCWs working under the umbrella of the PHCC during COVID-19 compared to previous year and uncovered the main associated factors. PHCC is the main provider of primary healthcare services in Qatar. It has played a vital role in containing the spread of COVID-19 infection [20, 21]. On 29 February 2020, Qatar confirmed the first COVID-19 case in a traveler who had returned from Iran [22]. It was only until March 6, 2020, when community transmission of the SARS-CoV-2 was documented among craft and manual workers living in crowded accommodations [23]. Since then, the number of cases increased dramatically reaching the peak of the first pandemic wave in mid-May 2020 [24].

Every single HCW was needed during the unprecedented crises of COVID-19. For this, Qatar has put the annual leaves on hold to deal with this national emergency while sick leaves were permitted. Despite this, we found that wave 1 had significantly less incidence of sick leaves per day per 1000 HCWs compared to pre-COVID-19 period which might reflect that HCWs were devoted and eager to help in the country’s fight against COVID-19. On the other hand, wave 2 had significantly higher incidence of sick leaves compared to both pre-COVID-19 and Wave 1 which can be explained by the detection of increasing numbers of Variants of Concern (VOC) (particularly B.1.351 and B.1.1.7 variants) that were associated with of increased transmissibility, severity, and reduced neutralization by convalescent sera. The surge of such variants was maximized during March 2021 [24]. This is further confirmed by the fact that the rate of sick leaves due to suspected of confirmed COVID-19 infection are significantly higher in wave 2 compared to wave 1.

In this study, the number of sick leaves per person among female HCWs was significantly higher than that of male HCWs which is consistent with existing evidence that has repeatedly shown substantial gender gaps in sickness absence which seems to be higher among women compared to men [25, 26]. This might be explained by gender differences in relation to different health issues including pregnancy, and the differences in attitudes towards sickness absence that are largely shaped by the gender stereotypes in the society which consider the traditional woman as weak and dependent and the traditional man as competitive and independent making women more prone to sickness absence than men [27]. Similarly, the number of sick leaves per person among locals were about two times the number among expatriate HCWs. This might be explained by the fact that locals compared to expatriates have more social connections with their relatives being in their own home country which can facilitate the spread of infections during social gatherings and higher incidence of sick leaves among them. Expectedly, physicians and nurses had lower sick leave per person rates compared to other professions as they served as the frontline HCWs and the first line of defense against COVID-19. Moreover, Evidence has shown a high prevalence of presenteeism or sickness presence among physicians as they tend to attend work while ill [28, 29].

The results of the study reveal that respiratory issues, back/neck pain, gastroenteritis, and COVID-19 infection were the most frequent causes of sick leaves among HCWs. The distribution of sick leave reasons was found to vary significantly between different time periods. One key findings was that the rates of respiratory-related sick leaves that were not labeled as suspected or confirmed cases of COVID-19 infection were significantly lower in all stages compared to the pre-COVID-19 stage. This could be due to the inappropriate linking of respiratory complaints/illnesses to SAR CoV2 infection and labeling them as suspected COVID-19. This highlights the importance of proper identification and diagnoses of respiratory illnesses. Furthermore, wave 2 and subsequent stage had significantly higher rates of sick leaves due to back/neck pain. This can be explained by the fact that back and neck symptoms are common manifestations of COVID-19 and long COVID-19 syndrome [30, 31]. A community-based case–control study [31] showed that 24.4% of COVID-19 survivors reported lower back pain, compared to 15.7% of people who hadn’t been infected, forecasting an upcoming wave of low back pain burden among the general population worldwide after the pandemic era. In addition, the prolonged working hours for long period of times during the national emergency experienced by HCWs may trigger multiple musculoskeletal symptoms and complaints.

## Conclusion

Overall and cause specific sick leave rates among HCWs varied significantly across different periods of the COVID-19 pandemic. Sick leaves rates among HCWs were lower during the first wave and higher in the second wave of COVID-19 pandemic compared to pre-COVID-19. Males, expatriates and physicians and nurses were significantly associated with lower number of sick leaves per person. This study provides valuable insights into the reasons behind sick leaves among HCWs. This information can be used to develop strategies to mitigate the impact of sick leaves on HCWs and improve their overall well-being. By addressing the root causes of sick leaves, it is possible to reduce the burden on HCWs and ensure their continued ability to provide essential care to those in need.

## Supplementary Information

**Additional file 1:** **Table S1.** Number of sick leaves availed in each month of 2019 by sociodemographic characteristics for sick leaves. **Table S2.** Number of sick leaves availed in each month of 2020 by sociodemographic characteristics and reason for sick leave. **Table S3.** Number of sick leaves availed in each month of 2021 by sociodemographic characteristics and reason for sick leave. **Table S4.** Number of sick leaves availed in different stages of COVID-19 pandemic by sociodemographic characteristics and reason for sick leave. **Table S5.** Duration of sick leaves availed by sociodemographic characteristics, reason for sick leave and different waves.

## Data Availability

Data are available upon reasonable request.
