# Supplementary material for: Sickness absenteeism among primary health care workers in Qatar before and during the COVID-19 pandemic
Source: J Occup Med Toxicol. 2023 Mar 16;18:3. doi: 10.1186/s12995-023-00369-3 (PMC10018637; doi:10.1186/s12995-023-00369-3)
Supplement: Supplementary file 1 — Additional file 1: Table S1. Number of sick leaves availed in each month of 2019 by sociodemographic characteristics for sick leaves. Table S2. Number of sick leaves availed in each month of 2020 by sociodemographic characteristics and reason for sick leave. Table S3. Number of sick leaves availed in each month of 2021 by sociodemographic characteristics and reason for sick leave. Table S4. Number of sick leaves availed in different stages of COVID-19 pandemic by sociodemographic characteristics and reason for sick leave. Table S5. Duration of sick leaves availed by sociodemographic characteristics, reason for sick leave and different waves. [file 12995_2023_369_MOESM1_ESM.docx]

| **Characteristics** | | **Year 2019 (12945)** | | | | | | | | | | | |
| --- | --- | --- | --- | --- | --- | --- | --- | --- | --- | --- | --- | --- | --- |
|  |  | **Jan (1066)** | **Feb (1015)** | **Mar (1097)** | **Apr (1064)** | **May (1025)** | **Jun (723)** | **Jul (944)** | **Aug (675)** | **Sep (1307)** | **Oct (1417)** | **Nov (1281)** | **Dec (1331)** |
|  |  | **No (%)** | **No (%)** | **No (%)** | **No (%)** | **No (%)** | **No (%)** | **No (%)** | **No (%)** | **No (%)** | **No (%)** | **No (%)** | **No (%)** |
| **Age categories** | Less than 30 | 279 (26.2) | 264 (26) | 261 (23.8) | 236 (22.2) | 253 (24.7) | 167 (23.1) | 250 (26.5) | 157 (23.3) | 265 (20.3) | 285 (20.1) | 267 (20.8) | 324 (24.3) |
|  | 30-39 | 423 (39.7) | 438 (43.2) | 459 (41.8) | 465 (43.7) | 430 (42.0) | 313 (43.3) | 361 (38.2) | 281 (41.6) | 550 (42.1) | 645 (45.5) | 579 (45.2) | 551 (41.4) |
|  | 40-49 | 240 (22.5) | 214 (21.1) | 247 (22.5) | 246 (23.1) | 245 (23.9) | 174 (24.1) | 206 (21.8) | 163 (24.1) | 332 (25.4) | 318 (22.4) | 291 (22.7) | 315 (23.7) |
|  | 50 or more | 124 (11.6) | 99 (9.8) | 130 (11.9) | 117 (11.0) | 97 (9.5) | 69 (9.5) | 127 (13.5) | 74 (11.0) | 160 (12.2) | 169 (11.9) | 144 (11.2) | 141 (10.6) |
| **Gender** | Female | 639 (59.9) | 557 (54.9) | 646 (58.9) | 606 (57.0) | 556 (54.2) | 378 (52.3) | 446 (47.2) | 348 (51.6) | 743 (56.8) | 791 (55.8) | 726 (56.7) | 734 (55.1) |
|  | Male | 427 (40.1) | 458 (45.1) | 451 (41.1) | 458 (43.0) | 469 (45.8) | 345 (47.7) | 498 (52.8) | 327 (48.4) | 564 (43.2) | 626 (44.2) | 555 (43.3) | 597 (44.9) |
| **Nationality*** | Expatriate | 778 (73.0) | 740 (72.9) | 803 (73.2) | 765 (71.9) | 750 (73.2) | 522 (72.2) | 727 (77.0) | 493 (73.0) | 938 (71.8) | 998 (70.4) | 945 (73.8) | 948 (71.2) |
|  | Local | 288 (27.0) | 275 (27.1) | 294 (26.8) | 299 (28.1) | 275 (26.8) | 201 (27.8) | 217 (23.0) | 182 (27.0) | 369 (28.2) | 419 (29.6) | 336 (26.2) | 383 (28.8) |
| **Profession** | Allied and Others | 674 (63.2) | 659 (64.9) | 719 (65.5) | 699 (65.7) | 705 (68.8) | 499 (69.0) | 678 (71.8) | 466 (69.0) | 863 (66) | 921 (65.0) | 824 (64.3) | 876 (65.8) |
|  | Nurse | 267 (25.0) | 240 (23.6) | 265 (24.2) | 256 (24.1) | 205 (20.0) | 155 (21.4) | 205 (21.7) | 157 (23.3) | 312 (23.9) | 368 (26.0) | 329 (25.7) | 333 (25) |
|  | Physician | 125 (11.7) | 116 (11.4) | 113 (10.3) | 109 (10.2) | 115 (11.2) | 69 (9.5) | 61 (6.5) | 52 (7.7) | 132 (10.1) | 128 (9.0) | 128 (10.0) | 122 (9.2) |
| **Employment type** | Contracted | 7 (0.7) | 3 (0.3) | 7 (0.6) | 9 (0.8) | 1 (0.1) | 6 (0.8) | 5 (0.5) | 7 (1.0) | 11 (0.8) | 13 (0.9) | 7 (0.5) | 11 (0.8) |
|  | Outsourced | 31 (2.9) | 30 (3.0) | 31 (2.8) | 26 (2.4) | 25 (2.4) | 21 (2.9) | 28 (3.0) | 10 (1.5) | 37 (2.8) | 43 (3.0) | 21 (1.6) | 41 (3.1) |
|  | Permanent | 1017 (95.4) | 972 (95.8) | 1043 (95.1) | 1017 (95.6) | 993 (96.9) | 685 (94.7) | 904 (95.8) | 654 (96.9) | 1247 (95.4) | 1348 (95.1) | 1238 (96.6) | 1261 (94.7) |
|  | Temporary | 11 (1.0) | 10 (1.0) | 16 (1.5) | 12 (1.1) | 6 (0.6) | 11 (1.5) | 7 (0.7) | 4 (0.6) | 12 (0.9) | 13 (0.9) | 15 (1.2) | 18 (1.4) |

Table S1: Number of sick leaves availed in each month of 2019 by sociodemographic characteristics for sick leaves.

*More than 70 nationalities were reported

Table S2: Number of sick leaves availed in each month of 2020 by sociodemographic characteristics and reason for sick leave.

| **Characteristics** | | **Year 2020 (13557)** | | | | | | | | | | | |
| --- | --- | --- | --- | --- | --- | --- | --- | --- | --- | --- | --- | --- | --- |
|  |  | **Jan (1465)** | **Feb (1488)** | **Mar (1563)** | **Apr (1069)** | **May (623)** | **Jun (1071)** | **Jul (949)** | **Aug (790)** | **Sep (1092)** | **Oct (1221)** | **Nov (1176)** | **Dec (1050)** |
|  |  | **No (%)** | **No (%)** | **No (%)** | **No (%)** | **No (%)** | **No (%)** | **No (%)** | **No (%)** | **No (%)** | **No (%)** | **No (%)** | **No (%)** |
| **Age categories** | Less than 30 | 380 (25.9) | 343 (23.1) | 364 (23.3) | 215 (20.1) | 151 (24.2) | 227 (21.2) | 210 (22.1) | 187 (23.7) | 238 (21.8) | 251 (20.6) | 214 (18.2) | 247 (23.5) |
|  | 30-39 | 597 (40.8) | 631 (42.4) | 686 (43.9) | 487 (45.6) | 294 (47.2) | 511 (47.7) | 458 (48.3) | 342 (43.3) | 472 (43.2) | 553 (45.3) | 558 (47.4) | 436 (41.5) |
|  | 40-49 | 307 (21) | 333 (22.4) | 353 (22.6) | 258 (24.1) | 128 (20.5) | 241 (22.5) | 204 (21.5) | 194 (24.6) | 275 (25.2) | 314 (25.7) | 293 (24.9) | 253 (24.1) |
|  | 50 or more | 181 (12.4) | 181 (12.2) | 160 (10.2) | 109 (10.2) | 50 (8.0) | 92 (8.6) | 77 (8.1) | 67 (8.5) | 107 (9.8) | 103 (8.4) | 111 (9.4) | 114 (10.9) |
| **Gender** | Female | 1044 (71.3) | 1104 (74.2) | 1193 (76.3) | 789 (73.8) | 431 (69.2) | 827 (77.2) | 763 (80.4) | 636 (80.5) | 829 (75.9) | 943 (77.2) | 909 (77.3) | 779 (74.2) |
|  | Male | 421 (28.7) | 384 (25.8) | 370 (23.7) | 280 (26.2) | 192 (30.8) | 244 (22.8) | 186 (19.6) | 154 (19.5) | 263 (24.1) | 278 (22.8) | 267 (22.7) | 271 (25.8) |
| **Nationality*** | Expatriate | 804 (54.9) | 786 (52.8) | 971 (62.1) | 756 (70.7) | 446 (71.6) | 719 (67.1) | 604 (63.6) | 468 (59.2) | 688 (63) | 815 (66.7) | 776 (66.0) | 645 (61.4) |
|  | Local | 661 (45.1) | 702 (47.2) | 592 (37.9) | 313 (29.3) | 177 (28.4) | 352 (32.9) | 345 (36.4) | 322 (40.8) | 404 (37) | 406 (33.3) | 400 (34.0) | 405 (38.6) |
| **Profession** | Allied and Others | 984 (67.2) | 994 (66.8) | 974 (62.3) | 557 (52.1) | 378 (60.7) | 617 (57.6) | 591 (62.3) | 494 (62.5) | 696 (63.7) | 753 (61.7) | 701 (59.6) | 654 (62.3) |
|  | Nurse | 348 (23.8) | 332 (22.3) | 428 (27.4) | 393 (36.8) | 201 (32.3) | 364 (34.0) | 275 (29) | 219 (27.7) | 276 (25.3) | 338 (27.7) | 350 (29.8) | 279 (26.6) |
|  | Physician | 133 (9.1) | 162 (10.9) | 161 (10.3) | 119 (11.1) | 44 (7.1) | 90 (8.4) | 83 (8.7) | 77 (9.7) | 120 (11) | 130 (10.6) | 125 (10.6) | 117 (11.1) |
| **Employment type** | Contracted | 9 (0.6) | 14 (0.9) | 25 (1.6) | 70 (6.5) | 60 (9.6) | 43 (4.0) | 37 (3.9) | 14 (1.8) | 40 (3.7) | 29 (2.4) | 49 (4.2) | 28 (2.7) |
|  | Outsourced | 33 (2.3) | 35 (2.4) | 50 (3.2) | 20 (1.9) | 18 (2.9) | 21 (2.0) | 30 (3.2) | 31 (3.9) | 42 (3.8) | 40 (3.3) | 23 (2.0) | 18 (1.7) |
|  | Permanent | 1407 (96) | 1427 (95.9) | 1476 (94.4) | 969 (90.6) | 523 (83.9) | 974 (90.9) | 855 (90.1) | 719 (91) | 966 (88.5) | 1102 (90.3) | 1064 (90.5) | 972 (92.6) |
|  | Temporary | 16 (1.1) | 12 (0.8) | 12 (0.8) | 10 (0.9) | 22 (3.5) | 33 (3.1) | 27 (2.8) | 26 (3.3) | 44 (4.0) | 50 (4.1) | 40 (3.4) | 32 (3.0) |
| **Reason for sick leave*** | COVID **^*^** | 0 (0.0) | 0 (0.0) | 33 (2.1) | 288 (26.9) | 203 (32.6) | 345 (32.2) | 179 (18.9) | 181 (22.9) | 247 (22.6) | 290 (23.8) | 241 (20.5) | 137 (13.0) |
|  | Non-COVID | 1465 (100.0) | 1488 (100.0) | 1530 (97.9) | 781 (73.1) | 420 (67.4) | 726 (67.8) | 770 (81.1) | 609 (77.1) | 845 (77.4) | 931 (76.2) | 935 (79.5) | 913 (87) |

*****Suspected or confirmed COVID-19 infection

Table S3: Number of sick leaves availed in each month of 2021 by sociodemographic characteristics and reason for sick leave

| **Characteristics** | | **Year 2021 (14630)** | | | | | | | |
| --- | --- | --- | --- | --- | --- | --- | --- | --- | --- |
|  |  | **Jan (1465)** | **Feb (1488)** | **Mar (1563)** | **Apr (1069)** | **May (623)** | **Jun (1071)** | **Jul (949)** | **Aug (790)** |
|  |  | **No (%)** | **No (%)** | **No (%)** | **No (%)** | **No (%)** | **No (%)** | **No (%)** | **No (%)** |
| **Age categories** | Less than 30 | 314 (22) | 396 (20.3) | 583 (20.7) | 515 (21.9) | 265 (22.2) | 450 (24) | 293 (24) | 422 (23.5) |
|  | 30-39 | 602 (42.2) | 835 (42.7) | 1178 (41.7) | 999 (42.6) | 494 (41.4) | 754 (40.3) | 500 (41) | 720 (40.1) |
|  | 40-49 | 349 (24.5) | 490 (25.1) | 718 (25.4) | 574 (24.5) | 279 (23.4) | 456 (24.4) | 299 (24.5) | 432 (24.1) |
|  | 50 or more | 161 (11.3) | 234 (12) | 343 (12.2) | 259 (11) | 156 (13.1) | 212 (11.3) | 127 (10.4) | 221 (12.3) |
| **Gender** | Female | 1072 (75.2) | 1461 (74.7) | 2107 (74.7) | 1815 (77.3) | 915 (76.6) | 1426 (76.2) | 891 (73.1) | 1323 (73.7) |
|  | Male | 354 (24.8) | 494 (25.3) | 715 (25.3) | 532 (22.7) | 279 (23.4) | 446 (23.8) | 328 (26.9) | 472 (26.3) |
| **Nationality*** | Expatriate | 908 (63.7) | 1166 (59.6) | 1655 (58.6) | 1383 (58.9) | 649 (54.4) | 969 (51.8) | 624 (51.2) | 873 (48.6) |
|  | Local | 518 (36.3) | 789 (40.4) | 1167 (41.4) | 964 (41.1) | 545 (45.6) | 903 (48.2) | 595 (48.8) | 922 (51.4) |
| **Profession** | Allied and Others | 888 (62.3) | 1233 (63.1) | 1791 (63.5) | 1505 (64.1) | 789 (66.1) | 1270 (67.8) | 854 (70.1) | 1260 (70.2) |
|  | Nurse | 357 (25) | 519 (26.5) | 742 (26.9) | 603 (25.7) | 266 (22.3) | 437 (23.3) | 284 (23.3) | 392 (21.8) |
|  | Physician | 181 (12.7) | 203 (10.4) | 289 (10.2) | 239 (10.2) | 139 (11.6) | 165 (8.8) | 81 (6.6) | 143 (8) |
| **Employment type** | Contracted | 28 (2) | 45 (2.3) | 39 (1.4) | 40 (1.7) | 25 (2.1) | 35 (1.9) | 32 (2.6) | 17 (0.9) |
|  | Outsourced | 26 (1.8) | 26 (1.3) | 45 (1.6) | 23 (1) | 9 (0.8) | 33 (1.8) | 16 (1.3) | 15 (0.8) |
|  | Permanent | 1325 (92.9) | 1819 (93) | 2646 (93.8) | 2207 (94) | 1127 (94.4) | 1771 (94.6) | 1140 (93.5) | 1712 (95.4) |
|  | Temporary | 47 (3.3) | 65 (3.3) | 92 (3.3) | 77 (3.3) | 33 (2.8) | 33 (1.7) | 31 (2.5) | 51 (3) |
| **Reason for sick leave*** | COVID **^*^** | 237 (16.6) | 337 (17.2) | 494 (17.5) | 520 (22.2) | 112 (9.4) | 144 (7.7) | 87 (7.1) | 175 (9.7) |
|  | Non-COVID | 1189 (83.4) | 1618 (82.8) | 2328 (82.5) | 1827 (77.8) | 1082 (90.6) | 1728 (92.3) | 1132 (92.9) | 1620 (90.3) |

.

Table S4: Number of sick leaves availed in different stages of COVID-19 pandemic by sociodemographic characteristics and reason for sick leave

| **Characteristics** | | **Pre-COVID Pandemic**  **(15898)** | **Wave 1**  **(7157)** | **Post Wave 1**  **(4873)** | **Wave 2**  **(7124)** | **Post Wave 2**  **(6080)** |
| --- | --- | --- | --- | --- | --- | --- |
|  |  | **No (%)** | **No (%)** | **No (%)** | **No (%)** | **No (%)** |
| **Age categories** | Less than 30 | 3731 (23.5) | 1592 (22.2) | 1026 (21.1) | 1494 (21) | 1430 (23.5) |
|  | 30-39 | 6723 (42.3) | 3250 (45.4) | 2149 (44.1) | 3012 (42.3) | 2468 (40.6) |
|  | 40-49 | 3631 (22.8) | 1653 (23.1) | 1209 (24.8) | 1782 (25) | 1466 (24.1) |
|  | 50 or more | 1813 (11.4) | 662 (9.2) | 489 (10) | 836 (11.7) | 716 (11.8) |
| **Gender** | Female | 8760 (55.1) | 5468 (76.4) | 3703 (76) | 5383 (75.6) | 4555 (74.9) |
|  | Male | 7138 (44.9) | 1689 (23.6) | 1170 (24) | 1741 (24.4) | 1525 (25.1) |
| **Nationality** | Expatriate | 11555 (72.7) | 4652 (65) | 3144 (64.5) | 4204 (59) | 3115 (51.2) |
|  | Local | 4343 (27.3) | 2505 (35) | 1729 (35.5) | 2920 (41) | 2965 (48.8) |
| **Profession** | Allied and Others | 10561 (66.4) | 4307 (60.2) | 2996 (61.5) | 4529 (63.6) | 4173 (68.6) |
|  | Nurse | 3772 (23.7) | 2156 (30.1) | 1324 (27.2) | 1864 (26.2) | 1379 (22.7) |
|  | Physician | 1565 (9.8) | 694 (9.7) | 553 (11.3) | 731 (10.3) | 528 (8.7) |
| **Employment Type** | Contracted | 110 (0.7) | 289 (4) | 134 (2.7) | 124 (1.7) | 109 (1.8) |
|  | Outsourced | 412 (2.6) | 212 (3) | 107 (2.2) | 94 (1.3) | 73 (1.2) |
|  | Permanent | 15213 (95.7) | 6482 (90.6) | 4463 (91.6) | 6672 (93.7) | 5750 (94.6) |
|  | Temporary | 163 (1) | 174 (2.4) | 169 (3.5) | 234 (3.3) | 148 (2.4) |

Pre-COVID Pandemic: January 2019 – February 2020

Wave 1: March 2020 – September 2020

Post wave 1: October 2020 – January 2021

Wave 2: February 2021 – April 2021

Post wave 2: May 2021 – August 2021

Table S5: Duration of sick leaves availed by sociodemographic characteristics, reason for sick leave and different waves.

| **Characteristics** | | **Average duration of sick leave (in days)** |
| --- | --- | --- |
| **Age categories** | Less than 30 | 1.64 |
|  | 30-39 | 1.80 |
|  | 40-49 | 1.81 |
|  | 50 or more | 1.82 |
| **Gender** | Female | 1.74 |
|  | Male | 1.86 |
| **Nationality*** | Expatriate | 1.90 |
|  | Local | 1.59 |
| **Profession** | Allied and Others | 1.69 |
|  | Nurse | 1.92 |
|  | Physician | 1.86 |
| **Reason for sick leave** | Respiratory related | 1.53 |
|  | Back and/or neck related | 1.40 |
|  | Gastroenteritis | 1.32 |
|  | Suspected or confirmed COVID-19 | 4.10 |
|  | Contact with confirmed or suspected COVID-19 case | 4.41 |
|  | Dental related | 1.38 |
|  | Pregnancy related | 1.53 |
|  | Mental related | 1.56 |
|  | Malaise, myalgia or fatigue | 1.38 |
|  | Tension type headache | 1.23 |
|  | Others | 1.53 |
| **Wave*** | Pre-COVID Pandemic | 1.34 |
|  | Wave 1 | 2.47 |
|  | Post Wave 1 | 1.87 |
|  | Wave 2 | 2.14 |
|  | Post Wave 2 | 1.54 |

*****Note that the average duration of leaves after excluding COVID-19 related leaves in the periods of Wave1, Post Wave 1, Wave 2 and Post Wave 2 are 1.85, 1.53, 1.54 and 1.38 respectively.
